# Transient remission of pre-existing left bundle branch block during general anesthesia in a centenarian

**DOI:** 10.1186/s40981-019-0288-0

**Published:** 2019-10-24

**Authors:** Takeaki Shiga, Takashi Suzuki, Masayuki Somei, Noriko Okumura

**Affiliations:** 1 Department of Anesthesiology, Showa University Koto Toyosu Hospital, 5-1-38, Toyosu, Koto-ku, Tokyo, 135-8577 Japan; 2 Department of Intensive Care Medicine, Showa University School of Medicine, 1-5-8 Hatanodai, Shinagawa-ku, Tokyo, 142-8666 Japan

**Keywords:** Left bundle branch block, Remission, Centenarian, General anesthesia, Hip fracture, Heart rate, Blood pressure

We present a case of a centenarian patient in whom pre-existing left bundle branch block (LBBB) transiently reverted to normal ventricular conduction during general anesthesia. A 104-year-old woman with a history of hypertension, chronic heart failure, and cognitive impairment was admitted for surgical repair of a femoral neck fracture. The standard 12-lead electrocardiogram (ECG) on admission revealed left axis deviation and complete LBBB with a heart rate (HR) of 60 bpm. Echocardiography indicated left ventricular dyssynchrony with an ejection fraction of 51%. Due to her restless and agitated behavior, general anesthesia was selected for surgery.

On arrival in the operating room, the patient’s blood pressure (BP) was 170/110 mmHg and HR was 110 bpm with a regular rhythm. ECG monitoring showed a wide QRS complex (140 ms) with RsrS pattern (Fig. 1a). Anesthesia was induced with fentanyl, remifentanil, propofol, and rocuronium, followed by insertion of a supraglottic airway, and maintained with desflurane, remifentanil, and fentanyl. Her lungs were mechanically ventilated. Twenty minutes after the commencement of anesthesia, the QRS complex abruptly narrowed to an rSr′ pattern (80 ms) with a HR of 80 bpm and BP of 100/50 mmHg (Fig. 1b). Surgery was commenced after femoral nerve block using levobupivacaine.

**Fig. 1:**
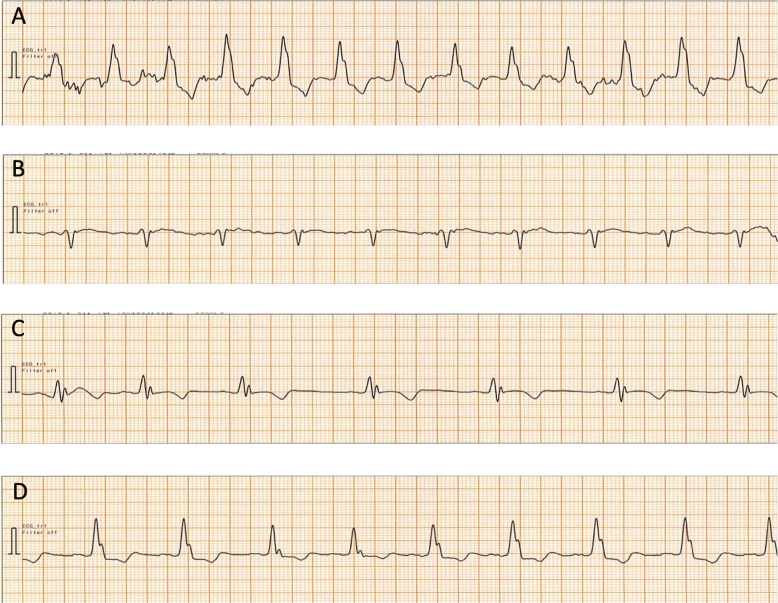
Chronological changes in QRS complex morphology recorded on a patient monitor (CM5) during anesthesia. **a** Immediately before anesthesia induction. **b** 20 min after the start of anesthesia. **c** End of surgery. **d** During extubation

Intraoperatively, HR, BP, S_P_O_2_, and end-tidal CO_2_ were maintained at 50–80 bpm, 90/40–120/60 mmHg, 99–100%, and 32–43 mmHg, respectively. At the end of the surgery that lasted for 33 min, the QRS complex widened to an RSr pattern (140 ms) at a HR of 50 bpm and BP of 100/60 mmHg (Fig. 1c) for a few minutes. Next, during the extubation phase, which was immediately after the reversal of neuromuscular block by sugammadex, QRS morphology changed to an RsrS pattern with the same QRS duration (140 ms). HR and BP at this time were 75 bpm and 170/100 mmHg (Fig. 1d). A 12-lead ECG performed 6 days postoperatively showed LBBB at a HR of 64 bpm, comparable to that in the preoperative period. The patient developed postoperative pneumonia requiring extended hospitalization and was discharged to a nursing home on the 28th postoperative day.

LBBB is commonly associated with structural heart disease and left ventricular dysfunction and is thought to increase cardiac mortality in patients with congestive heart failure [1]. LBBB can occur in a transient or intermittent manner, developing under diverse clinical settings, including during anesthesia, due to various etiologies such as blood pressure perturbation, tachycardia, and bradycardia. Conversely, the episodic disappearance of LBBB during anesthesia has rarely been reported [2–4]. The presented case shares similarities with three such previously reported cases, as summarized in Table 1. The disappearance of the LBBB shortly after induction of anesthesia and the reappearance during or immediately after emergence from anesthesia are mostly common characteristics of these cases with a partially exceptional case reported by Garcia et al. These cases suggest that sufficient vasodilatory effects of volatile anesthetics and negative chronotropic effects of narcotics might contribute to remission.

**Table 1:** Previously reported cases of transient remission of pre-existing left bundle branch block during general anesthesia

References	Age/sex	Comorbidities	Surgical procedure	Induction agents	Maintenance agents	Elapsed time from anesthesia induction to LBBB remission (min)	Situation at LBBB reappearance	Suspected cause of remission
Garcia et al. (1997) [2]	58/M	Hypertension	Inguinal hernia repair	Thiopental	Enflurane	15	Sustained remission*	Blood pressure reduction
				Fentanyl	Nitrous oxide			
				Suxamethonium	Atracurium			
Mishra et al. (2009) [3]	45/F	Hypertension	Mastectomy	Propofol	Isoflurane	25^†^	On reversal of neuromuscular block	Heart rate reduction (< 60 bpm)
				Fentanyl				
					Nitrous oxide			
				Vecuronium				
Silva et al. (2017) [4]	73/F	Hypertension, bronchial asthma, diabetes mellitus (type II)	Exploratory laparotomy	Propofol	Sevoflurane	30^‡^	On arrival in the PACU	Heart rate reduction (< 75 bpm)
				Remifentanil				
					Remifentanil			
				Rocuronium				
Present case	104/F	Hypertension, chronic heart failure, cognitive impairment	Femoral neck fracture fixation	Propofol	Desflurane	20	During the extubation period	Blood pressure reduction
				Remifentanil				
				Fentanyl	Remifentanil			
				Rocuronium				

*LBBB* left bundle branch block, *M* male, *F* female, *PACU* postanesthesia care unit

*Sustained remission was reconfirmed 1 month postoperatively

^†^LBBB disappeared again for 10 min during postoperative observation for 1 h

^‡^Bronchospasm occurred during induction of anesthesia and was successfully treated with inhalation of salbutamol and ipratropium

The choice of anesthesia technique for hip fracture fixation in elderly patients has been discussed [5]. We speculate, although with our limited experience and from previous reports, that appropriate general anesthesia possibly has positive impacts on ventricular conduction delay in even centenarian patients with LBBB.

## Data Availability

Please contact the corresponding author to request data access.

## Abbreviations

ECG: Electrocardiogram
HR: Heart rate
BP: Blood pressure
LBBB: Left bundle branch block
SpO_2_: Arterial oxygen saturation
